# The complex relationship between human immunodeficiency virus infection and death in adults being treated for tuberculosis in Cape Town, South Africa

**DOI:** 10.1186/s12889-015-1914-z

**Published:** 2015-06-18

**Authors:** Muhammad Osman, James A. Seddon, Rory Dunbar, Heather R. Draper, Carl Lombard, Nulda Beyers

**Affiliations:** City of Cape Town Health Directorate, Cape Town, South Africa; Department of Paediatric Infectious Diseases, Imperial College London, Norfolk Place, London, W2 1PG UK; Desmond Tutu TB Centre, Department of Paediatrics and Child Health, Faculty of Medicine and Health Sciences, Stellenbosch University, Tygerberg, South Africa; Biostatistics Unit, Medical Research Council, Tygerberg, South Africa

**Keywords:** Tuberculosis, Death, Adults, HIV, Cape Town

## Abstract

**Background:**

Despite recognised treatment strategies, mortality associated with tuberculosis (TB) remains significant. Risk factors for death during TB treatment have been described but the complex relationship between TB and HIV has not been fully understood.

**Methods:**

A retrospective analysis of all deaths occurring during TB treatment in Cape Town, South Africa between 2009 and 2012 were done to investigate risk factors associated with this outcome. The main risk factor was HIV status at the start of treatment and its interaction with age, sex and other risk factors were evaluated using a binomial regression model and thus relative risks (RR) are reported.

**Results:**

Overall in the 93,133 cases included in the study 4619 deaths (5 %) were recorded. Across all age groups HIV-positive patients were more than twice as likely to die as HIV-negative patients, RR = 2.19 (95 % CI: 2.03–2.37). However in an age specific analysis HIV-positive patients 15–24 and 25–34 years old were at an even higher risk of dying than HIV-negative patients, RR = 4.82 and RR = 3.76 respectively. Gender also modified the effect of HIV- with positive women having a higher risk of death than positive men, RR = 2.74 and RR = 1.94 respectively.

**Conclusion:**

HIV carries an increased risk of death in this study but specific high-risk groups pertaining to the impact of HIV are identified. Innovative strategies to manage these high risk groups may contribute to reduction in HIV-associated death in TB patients.

## Background

Despite effective chemotherapy, tuberculosis (TB)-associated mortality remains high and is the leading cause of death in South Africa accounting for 11 % of all South African deaths [1]. The World Health Organization (WHO) estimated 1.4 million TB deaths in 2012, of which 430 000 were among Human Immunodeficiency Virus (HIV)-positive individuals [2].

Waitt and Squire identified 62 studies in a systematic review to explore risk factors for death in patients started on TB treatment [3]. Retrospective and prospective cohorts, case control studies and cross sectional studies were included. This review revealed two distinct epidemics with separate risk factors for death. Risk factors for death while on TB treatment in high HIV/TB regions include HIV positivity, atypical chest radiographic features, sputum smear-negative disease, advanced immunosuppression and malnutrition and in low HIV/TB burden regions include increased age, typical features of severe TB on chest radiograph, smear positivity and socio-demographic disadvantage (alcohol, injectable drug use and homelessness). It is possible, even likely, that these two epidemics co-exist within populations. HIV positivity and severe immunosuppression are risk factors for the development of TB disease and for death on TB treatment [4, 5]. Combination antiretroviral therapy (cART) offers protection both from the development of TB [6] and from death while on TB treatment [7]. However, the picture is more complicated and it is still unclear why some patients (both HIV-positive and -negative) die following the diagnosis of TB and exactly which HIV-positive patients are at the greatest risk.

Understanding factors associated with death while on TB treatment permits the development of strategies to strengthen the TB control programme. We aimed to determine risk factors for death in adult patients on TB treatment under programmatic conditions in Cape Town and describe these factors in relation to HIV infection.

## Methods

### Setting

This retrospective cohort study was conducted in the City of Cape Town district (population estimate of 3.7 million in 2011 [1]), located in the Western Cape of South Africa. There are 160 primary health care (PHC) facilities (clinics, mobile/satellite facilities and community health centres) in the district which offer free TB testing, of which 103 offer outpatient TB treatment. In addition there are 15 hospitals where inpatients can be started on treatment and then referred to PHC facilities for continuation of treatment. These PHC TB treatment facilities are distributed across the city and organised into eight sub-districts.

The City of Cape Town district reported a decrease in reported TB cases per population from 877/100,000 in 2009 to 663/100,000 in 2013 with a cure rate of 83 % among all patients who started TB treatment in 2013 [8]. Laboratory diagnosis of TB in Cape Town utilised smear microscopy, culture and line probe assay until August 2011, after which the GeneXpert MTB/RIF assay (Xpert; Cepheid, Sunnyvale, CA) was progressively introduced for the testing of all patients with presumptive TB. TB treatment regimens were in accordance with international standards: combination chemotherapy with rifampicin, isoniazid, ethambutol and pyrazinamide; an initial intensive phase followed by a continuation phase. HIV testing was performed using rapid testing according to National guidelines [9].

### Study population and data sources

All TB patients treated for drug-susceptible TB are recorded in a paper-based register at their PHC treatment facility. If started on treatment in hospital, they should still be recorded at their local PHC facility. These paper registers are captured at a sub-district level into an electronic database, (ETR.net) which includes patient-specific details (age, gender, address), disease-specific details (site of disease, sputum smear results, treatment regimen, HIV status and CD4 count) and TB outcome (treatment completed, cured, defaulted, died, failed). All adults (≥15 years) treated for drug-susceptible TB in the City of Cape Town District, and recorded in the ETR.net, were included if started on treatment between 1 January 2009 and 30 June 2012. This included adults with any form of TB but excluded cases with confirmed drug-resistant TB.

### Definitions

New cases were those recorded in ETR.net as not having been treated previously for TB or who had received less than four weeks of TB treatment. Retreatment cases were those recorded in ETR.net as having received more than four weeks of TB treatment, regardless of time since the previous episode or outcome. Patients were recorded as sputum smear-positive if a sputum specimen, taken prior to TB treatment, was noted to be 1+, 2+ or 3 + for acid fast bacilli on microscopy. Patients were considered HIV-positive if, in ETR.net, they had a positive HIV status recorded, were recorded to be on antiretroviral medication, if they had a recorded CD4 result or if they were on co-trimoxazole. TB treatment outcomes were those recorded in the ETR.net.

### Data collection

Data for all adult TB cases were exported from ETR.net per sub-district and combined into a single database. Personal identifiers including first name, surname and date of birth were included for matching and exclusion of duplicate entries, and subsequently removed. The final database contained no personal identifiers. The data was analysed using Stata 12.1 (StataCorp. 2011. *Stata Statistical Software: Release 12.* College Station, TX: StataCorp LP). Ethical approval and a waiver of individual informed consent were received from the Stellenbosch University Health Research Ethics Committee (S12/01/018) and permission was obtained from the City of Cape Town Health Directorate.

### Statistical analysis

The proportion of patients dying was displayed by HIV status for each of the demographic and clinical variables. All variables were analysed categorically using frequencies and percentages. Age was stratified: 15–24 years, 25–34 years, 35–44 years, 45–54 years, 55–64 years and 65+ years and CD4 count was categorized: <50 cells/mm^3^, 51–200 cells/mm^3^, 201–350 cells/mm^3^, 351–500 cells/mm^3^ and >500 cells/mm^3^. Kaplan Meier curves were generated for time to death by HIV status. Time to death was calculated as the time in days between the TB treatment initiation date and the date of death documented in the ETR.net. Patients were censored from analysis either 180 days after TB initiation or at the time of their death, whichever event occurred first. A binomial log-linear regression model was used to determine which demographic and clinical characteristics were associated with death. Relative risks (RRs) and 95 % confidence intervals (CIs) were reported. Two- and three-way interactions between HIV and demographic and clinical characteristics were assessed and, if any interactions were statistically significant, the modified HIV mortality RRs were reported. A two-way interaction between CD4 count and age was assessed and the modified mortality RRs were reported. Standard errors for both binomial log-linear regression models were adjusted to account for any possible health care facility clustering.

## Results

### All TB cases

**Fig. 1:**
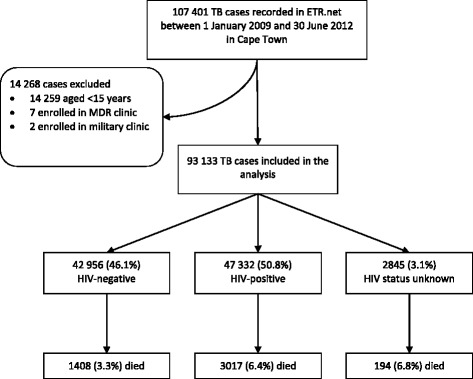
Schematic diagram of study population selected for analysis

Between 1 January 2009 and 30 June 2012, 107 401 TB cases were recorded in the ETR.net (Fig. 1). Of these, 14 259 were children less than 15 years of age, seven were cases of MDR-TB and two were enrolled at a military clinic; these were all excluded from further analysis. Demographic details as well as baseline TB data were available for analysis in the 93 133 TB cases. Sputum smear results were recorded in 89.9 % of all cases; HIV status could be determined in 96.9 % of TB cases and of those HIV-positive, 94.6 % had a CD4 count recorded. Of the 93 133 TB cases, 51 322 (55.1 %) were male, 47 332 (50.8 %) were HIV-positive and 4619 (5.0 %) died (Table 1).

**Table 1:** Demographic and clinical characteristics for adult TB cases, stratified by HIV status and death (*n* = 93 133)

	HIV-positive		HIV-negative		HIV status unknown		Total died (*n* = 4619)
	Alive	Died	Alive	Died	Alive	Dead	
	(*n* = 44 315)	(*n* = 3017)	(*n* = 41 548)	(*n* = 1408)	(*n* = 2651)	(*n* = 194)	
Mean age in years at registration (SD)	34.9 (9.1)	37.9 (10.3)	36.0 (14.4)	51.1 (15.8)	36.0 (14.5)	50.1 (17.4)	42.4 (14.0)
Age in years at registration							
15–24 (%)	4531 (95.6)	208 (4.4)	11 358 (99.1)	98 (0.9)	597 (97.5)	15 (2.5)	321 (1.9)
25–34 (%)	19 064 (94.8)	1044 (5.2)	10 292 (98.7)	134 (1.3)	857 (96.6)	30 (3.4)	1208 (3.8)
35–44 (%)	14 236 (93.2)	1035 (6.8)	7917 (97.6)	194 (2.4)	534 (94.5)	31 (5.5)	1260 (5.3)
45–54 (%)	5096 (90.8)	519 (9.2)	7122 (95.0)	377 (5.0)	343 (90.0)	38 (10.0)	934 (6.9)
55–64 (%)	1220 (88.0)	166 (12.0)	3383 (91.1)	329 (8.9)	182 (84.7)	33 (15.3)	528 (9.9)
> 64 (%)	168 (78.9)	45 (21.1)	1476 (84.2)	276 (15.8)	138 (74.6)	47 (25.4)	368 (17.1)
Gender							
Male (%)	21 037 (93.3)	1464 (6.8)	26 009 (96.3)	988 (3.7)	1709 (93.7)	115 (6.3)	2567 (5.1)
Female (%)	24 078 (93.9)	1553 (6.1)	15 539 (97.4)	420 (2.6)	942 (92.3)	79 (7.7)	2052 (4.8)
Median CD4 count for HIV-positive patients [IQR]	167 [77–298]	93 [37–191]					
< 50 cells/mm^3^ (%)	7039 (89.0)	872 (11.0)					
51–200 cells/mm^3^ (%)	17 671 (93.6)	1209 (6.4)					
201–350 cells/mm^3^ (%)	9895 (96.0)	407 (4.0)					
351–500 cells/mm^3^ (%)	4736 (96.9)	154 (3.1)					
> 500 cells/mm^3^ (%)	3171 (97.2)	91 (2.8)					
Missing	1803 (86.4)	284 (13.6)					
Baseline sputum smear							
Negative (%)	24 855 (93.7)	1591 (6.3)	13 590 (95.6)	628 (4.4)	884 (92.8)	69 (7.2)	2288 (5.6)
Positive (%)	14 572 (94.0)	938 (6.0)	25 392 (97.6)	625 (2.4)	1488 (95.3)	74 (4.7)	1637 (3.8)
No smear (%)	5888 (92.3)	488 (7.7)	2566 (94.3)	155 (5.7)	279 (84.5)	51 (15.5)	694 (7.4)
Disease classification							
PTB (%)	33 664 (93.9)	2205 (6.1)	37 132 (96.9)	1174 (3.1)	2293 (93.7)	155 (6.3)	3534 (4.6)
EPTB (%)	9410 (93.0)	705 (7.0)	4056 (95.1)	208 (4.9)	329 (90.6)	34 (9.4)	947 (6.4)
Both (%)	1241 (92.1)	107 (7.9)	360 (93.3)	26 (6.7)	29 (85.3)	5 (14.7)	138 (7.8)
Category							
Retreatment (%)	13 738 (92.2)	1167 (7.8)	11 520 (95.2)	582 (4.8)	637 (91.4)	60 (8.6)	1809 (6.5)
New (%)	30 577 (94.3)	1850 (5.7)	30 028 (97.3)	826 (2.7)	2014 (93.8)	134 (6.2)	2810 (4.3)
Registration year							
2009	12 771 (93.0)	965 (7.0)	11 506 (96.3)	436 (3.7)	1262 (93.4)	89 (6.6)	1490 (5.5)
2010	12 971 (93.6)	893 (6.4)	12 147 (96.7)	414 (3.3)	710 (93.7)	48 (6.3)	1355 (5.0)
2011	12 739 (94.2)	787 (5.8)	11 929 (97.0)	365 (3.0)	453 (91.5)	42 (8.5)	1194 (4.5)
2012	5834 (94.0)	372 (6.0)	5966 (96.9)	193 (3.1)	226 (93.8)	15 (6.2)	580 (4.6)

*SD* standard deviation, *IQR* interquartile range, *TB* tuberculosis, *PTB* pulmonary TB, *EPTB* extra-pulmonary TB

**Fig. 2:**
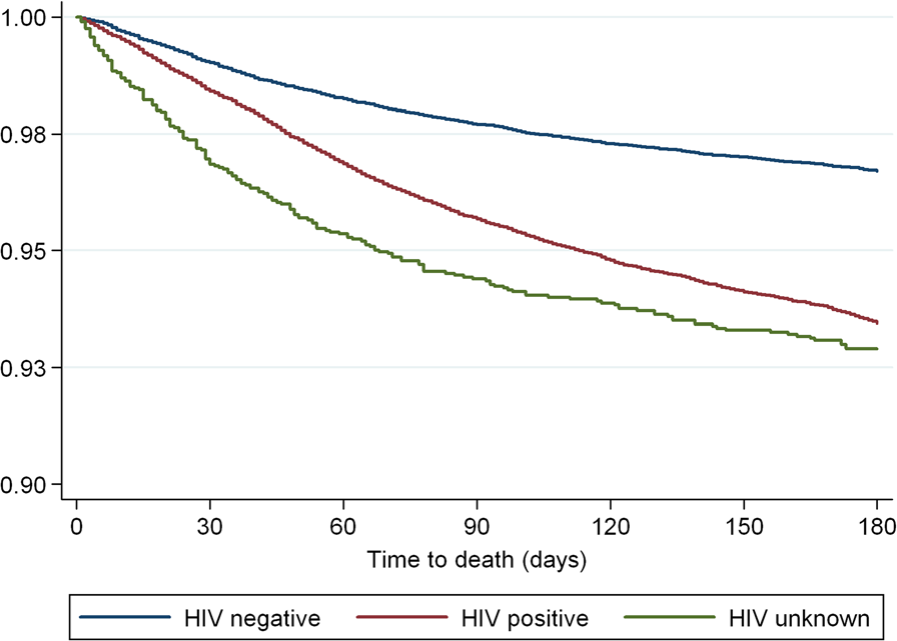
Kaplan-Meier survival curves depicting time to death according to HIV

TB cases who were HIV-positive (RR: 2.19; 95 % CI: 2.03, 2.37) or had an unknown HIV status (RR: 1.86; 95 % CI 1.57, 2.21) were at an increased risk of death (Table 2). Cases over 65 years were at higher risk of death than younger cases (RR: 9.16; 95 % CI 7.79, 10.80), those with extrapulmonary TB had a greater risk of death than those with pulmonary TB (RR: 1.14; 95 % CI: 1.04, 1.26) while cases with sputum smear-positive TB had a lower risk (RR: 0.88; 95 % CI: 0.82, 0.94) than those with sputum smear-negative TB. The risk of death was marginally higher in men than women (RR: 1.06; 95 % CI: 1.01, 1.12) and higher in retreatment cases (RR: 1.33; 95 % CI: 1.23, 1.45) (Table 2). The death rate fell between 2009 and 2012 (RR: 0.83; 95 % CI: 0.74, 0.93). Figure 2 displays the Kaplan-Meier survival curves showing the time to death by HIV status. Patients with unknown HIV status had more early deaths but the curve adopted a similar shape to the HIV-negative population after 90 days.

**Table 2:** Estimated RR of TB mortality from a main effects model using binomial log-linear regression (*n* = 93 133)

	RR	(95 % CI)
HIV status		
Negative	Ref	
Positive	2.19	(2.03–2.37)
Unknown	1.86	(1.57–2.21)
Age group		
15–24 years	Ref	
25–34 years	1.44	(1.28–1.63)
35–44 years	1.93	(1.70–2.18)
45–54 years	2.95	(2.64–3.30)
55–64 years	4.75	(4.12–5.48)
65+ years	9.16	(7.79–10.78)
Baseline smear		
Negative	Ref	
Positive	0.88	(0.82–0.94)
No smear	1.25	(1.13–1.37)
Disease classification		
PTB	Ref	
EPTB	1.14	(1.04–1.26)
Both	1.30	(1.10–1.55)
Gender		
Female	Ref	
Male	1.06	(1.01–1.12)
Type of treatment		
New	Ref	
Retreatment	1.34	(1.24–1.44)
Year		
2009	Ref	
2010	0.92	(0.86–0.99)
2011	0.83	(0.76–0.90)
2012^a^	0.83	(0.74–0.93)

*RR* relative risk, *CI* confidence interval, *TB* tuberculosis, *PTB* pulmonary TB, *EPTB* extra-pulmonary TB

^a^Only the first 6 months of 2012 were included in this analysis

### Impact of HIV

**Fig. 3:**
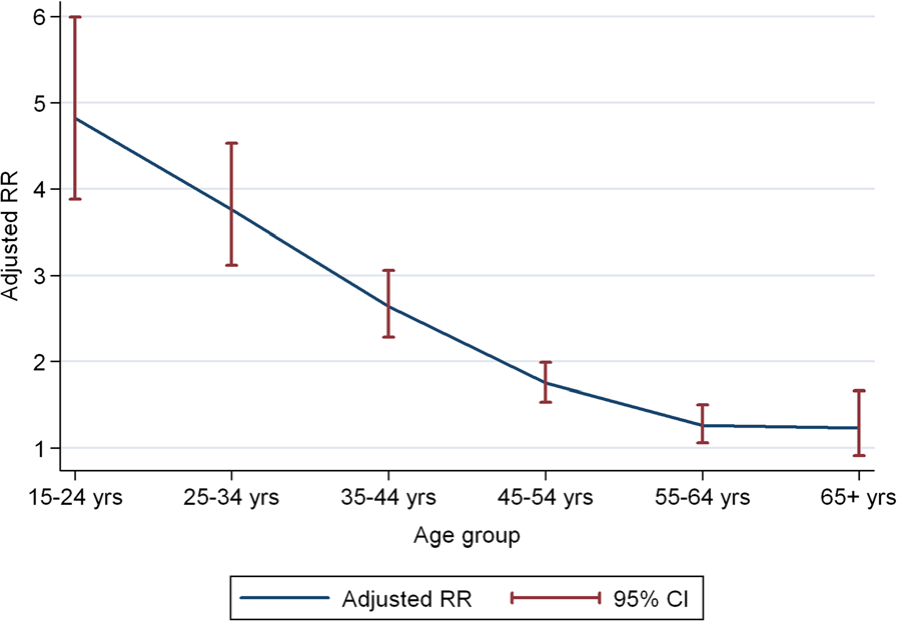
Relative risk estimates for death by age groups, comparing HIV-positive TB cases with HIV-negative cases

For 15–24 year olds, HIV-positive patients were nearly five times more likely to die than HIV-negative patients (RR: 4.82; 95 % CI: 3.88, 5.99), whereas for patients 55–64 years, HIV-positive patients were only marginally more likely to die (RR: 1.26; 95 % CI: 1.06, 1.50; Table 3 and Fig. 3). For individuals with sputum smear-positive disease, those with HIV were more likely to die than those without HIV (RR: 2.82; 95 % CI: 2.58, 3.07). This is greater than the increased risk in HIV-positive compared to HIV-negative patients with smear-negative disease (RR: 1.85; 95 % CI: 1.68, 2.04). HIV-positive women were more likely to die than HIV-negative women (RR: 2.74; 95 % CI: 2.43, 3.08). The effect of HIV was less pronounced in men (RR: 1.94; 95 % CI: 1.77, 2.11; Table 3).

**Table 3:** Crude and specific relative risk estimates for impact of HIV, comparing HIV-positive and negative patients

	RR	(95 % CI)	p-value
HIV Status			
HIV-positive	2.19	(2.03–2.37)	<0.001
Age group			
15–24 years	4.82	(3.88–5.99)	<0.001
25–34 years	3.76	(3.12–4.53)	<0.001
35–44 years	2.64	(2.28–3.06)	<0.001
45–54 years	1.75	(1.53–1.99)	<0.001
55–64 years	1.26	(1.06–1.50)	0.008
65+ years	1.23	(0.91–1.66)	0.169
Baseline smear			
Negative	1.85	(1.68–2.04)	<0.001
Positive	2.82	(2.58–3.07)	<0.001
No smear	1.77	(1.46–2.14)	<0.001
Gender			
Female	2.74	(2.43–3.08)	<0.001
Male	1.94	(1.77–2.11)	<0.001

*RR* relative risk, *CI* confidence interval

**Fig. 4:**
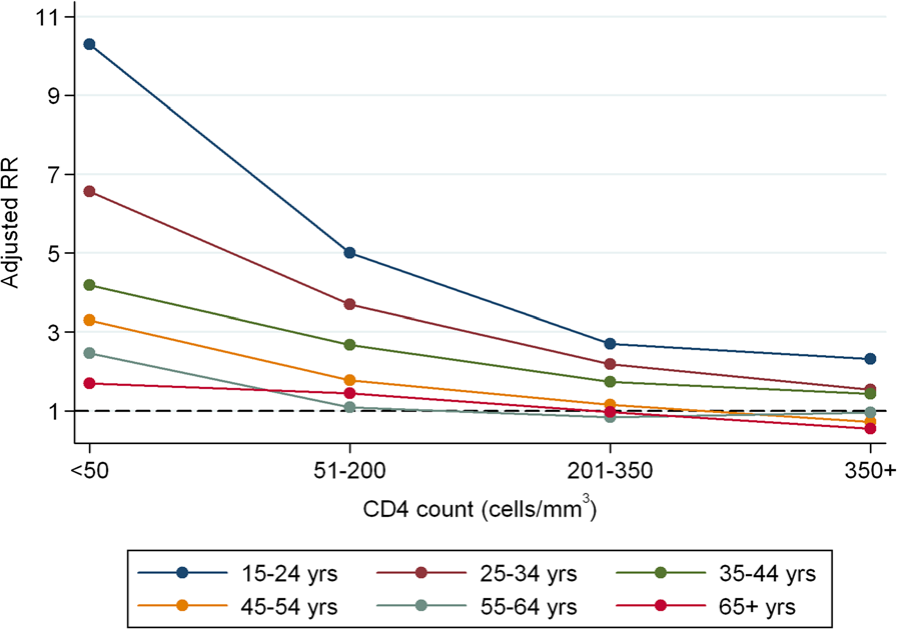
Relative risk for death between HIV-negative and-positive patients at different CD4 counts, stratified by age

In sub-group analysis, HIV-positive patients with CD4 count <50 cells/mm^3^ had high risk of death compared to HIV-negative patients (RR: 3.83; 95 % CI: 3.48, 4.21). HIV-positive patients with CD4 count above 350cells/mm^3^ had a similar risk of death compared to HIV-negative patients (RR: 1.07; 95 % CI: 0.93, 1.23; Table 4). In further sub-group analysis of HIV-positive TB cases by CD4 count and age, individuals 15–24 years with a CD4 count <50 cells/mm^3^ had high risk of death, compared to HIV-negative cases (RR: 10.29; 95 % CI: 7.66, 13.82), while older patients (55–64 years) with a CD4 count of <50 cells/mm^3^ had only a marginally increased risk (RR 2.46; 95 % CI: 1.88, 3.22; Table 4 and Fig. 4).

**Table 4:** Adjusted risk of death for HIV-positive patients by CD4 count compared to HIV-negative (*n* = 88 201) (excludes HIV unknown and CD4 missing)

	RR	(95 % CI)	p-value
All ages by CD4 count			
< 50 cells/mm^3^	3.83	(3.48–4.21)	<0.001
51–200 cells/mm^3^	2.21	(2.02–2.43)	<0.001
201–350 cells/mm^3^	1.39	(1.25–1.55)	<0.001
> 350 cells/mm^3^	1.07	(0.93–1.23)	0.321
Age 15–24 years by CD4 count			
< 50 cells/mm^3^	10.29	(7.66–13.8)	<0.001
51–200 cells/mm^3^	5.00	(3.74–6.68)	<0.001
201–350 cells/mm^3^	2.70	(1.84–3.96)	<0.001
> 350 cells/mm^3^	2.32	(1.59–3.39)	<0.001
Age 25–34 years by CD4 count			
< 50 cells/mm^3^	6.56	(5.41–7.95)	<0.001
51–200 cells/mm^3^	3.69	(3.05–4.47)	<0.001
201–350 cells/mm^3^	2.18	(1.73–2.74)	<0.001
> 350 cells/mm^3^	1.54	(1.15–2.06)	0.003
Age 35–44 years by CD4 count			
< 50 cells/mm^3^	4.18	(3.49–5.00)	<0.001
51–200 cells/mm^3^	2.67	(2.25–3.17)	<0.001
201–350 cells/mm^3^	1.74	(1.43–2.11)	<0.001
> 350 cells/mm^3^	1.43	(1.11–1.83)	0.005
Age 45–54 years by CD4 count			
< 50 cells/mm^3^	3.30	(2.75–3.96)	<0.001
51–200 cells/mm^3^	1.78	(1.55–2.05)	<0.001
201–350 cells/mm^3^	1.15	(0.91–1.45)	0.239
> 350 cells/mm^3^	0.72	(0.49–1.06)	0.099
Age 55–64 years by CD4 count			
< 50 cells/mm^3^	2.46	(1.88–3.22)	<0.001
51–200 cells/mm^3^	1.09	(0.78–1.51)	0.621
201–350 cells/mm^3^	0.84	(0.59–1.19)	0.329
> 350 cells/mm^3^	0.95	(0.63–1.43)	0.811
Age 65+ years by CD4 count			
< 50 cells/mm^3^	1.70	(1.03–2.80)	0.039
51–200 cells/mm^3^	1.45	(0.93–2.26)	0.101
201–350 cells/mm^3^	0.97	(0.58–1.61)	0.892
> 350 cells/mm^3^	0.55	(0.20–1.51)	0.247

*RR* relative risk, *CI* confidence interval

## Discussion

In this retrospective analysis we have shown HIV-positive and HIV-unknown patients with TB have an increased risk of death during TB treatment. We have demonstrated that older age contributes to an increase in relative risk of death independent of HIV status, a finding consistent with previous studies [10]. This may be due to concomitant pathology, less physical reserves or it may reflect an increasing frequency of non-TB causes of death. We have showed that the impact of HIV was greater in younger, as opposed to older, patients, with severe immunosuppression also having a greater impact on death in younger patients. These differences may reflect a combination of HIV greatly impacting on mortality in younger people combined with limited impact in those older. Older people frequently have additional pathologies that might affect their outcome for TB, in addition to HIV. Younger people, however, rarely have other conditions. Younger people may delay seeking care for their HIV more than older people which might impact on survival. Another possibility is that HIV-positive individuals who survive to older age are likely to have better controlled HIV and be taking cART conscientiously. The effect of gender has been described in a previous small study [11] and we have shown that men have a higher proportion of deaths on TB treatment than women regardless of HIV infection. However, we also demonstrated that the effect of HIV on death during TB treatment was greater in females; the reasons for this need further investigation.

The relationship between HIV positivity and both smear status and site of TB is also complex; previous studies have shown a relationship between sputum smear-negative pulmonary disease and mortality, as well as extra-pulmonary TB and mortality [12]. However in our study, the impact of HIV on death was greater in those who had smear-positive disease as opposed to smear-negative. Again, the reasons are unclear. Patients with unknown HIV status comprised a very small proportion (3.1 %) of the patients on TB treatment, reflecting excellent uptake of voluntary testing for HIV in this high risk population. Our Kaplan-Meier curve shows that some of those with unknown HIV status die rapidly, after which the curve assumes the trajectory of the HIV-negative cohort.

In an attempt to understand these interactions, it is important to consider factors which impact on mortality in patients treated for TB. Alcohol is strongly associated with the development of TB [13] and also with poor outcome on treatment [14, 15]. This is significant in Cape Town where there is a high rate of alcohol dependence and a complex interplay exists between the biomedical and social aspects of TB, HIV and alcohol. This may impact on the age and gender interactions with HIV and death. Smoking cigarettes and using solid fuels to cook and heat homes is also associated with TB, both the risk of developing TB as well as dying on treatment [16, 17]. This is also relevant in Cape Town where many, especially the young, smoke cigarettes, some smoke drugs and many still use solid fuels for cooking and heating. Diabetes [18, 19], malnutrition [11, 20–22] and mental health [23, 24] all affect both HIV and TB. These conditions are associated with or pre-dispose to TB and lead to poor outcomes. However, all three also have a relationship with HIV which can affect the endocrine system of individuals, as well as their nutrition and their psychological well-being. Patients with drug-resistant strains of *M. tuberculosis* have poorer outcomes than those with drug-susceptible strains [25], and although patients with known drug-resistant TB were not included in this analysis, it is likely that some cases would have been recorded in ETR.net. Although there does not appear to be a relationship between HIV and the occurrence of TB drug resistance [26], mortality is higher in HIV-positive patients with drug-resistant TB as opposed to HIV-negative patients with drug-resistant forms of disease [27].

Within the group of HIV-positive patients, there are additional risk factors for death. The degree of immunosuppression is associated with both the development of TB as well as poor outcomes if TB develops [5]. Not being on cART at the start of TB treatment, or not starting cART following the initiation of TB treatment, is associated with death [6]. Even for those on cART, with full virological suppression, a failure to develop immunological recovery has been shown to be associated with death [28]. The presence of opportunistic infections, in addition to TB, has also been shown to influence mortality [29]; co-infection with cryptococcus [30] and human T cell lymphotropic virus [31] has been associated with death in HIV-positive patients treated for TB. These factors may all contribute to interaction between age, gender, HIV and outcome. Studies from the pre-cART era [32], as well as studies following the introduction of cART [33], demonstrate a survival advantage in patients who had been given co-trimoxazole, suggesting the prevention of deaths from causes other than TB.

This study is one of the largest described cohorts of adults treated for TB. From this dataset we are able to tease out some of the intricacies of the relationship between HIV and TB. However, as with any retrospective study of routine data, limitations exist. First, missing data did occur. Although the dataset was relatively complete, the documentation of cART uptake was relatively poor and was not included in any descriptive outputs or any models. Although the number of patients with unknown HIV status was relatively small, the proportion was greater for older age-groups as compared to younger age-groups. It is possible that this may have influenced the perceived impact of HIV on death in different age-groups. There was some uncertainty around the accuracy of the recorded date of death and aside from the Kaplan Meier curve, we did not use this in our analysis. Some important variables such as smoking, alcohol use, opportunistic infections, body mass index etc. are not routinely recorded in ETR.net and require prospective studies for evaluation. The study defined death as a TB case dying before the end of treatment regardless of cause. It does not reflect the true mortality associated with TB, as it includes death due to accidents or co-morbidities. In HIV-positive populations, a significant proportion of deaths on TB treatment are not caused by TB [34, 35]. It also does not account for deaths occurring prior to treatment initiation, deaths in those who have defaulted or death occurring after treatment. As the study took place in one location, it may not be possible to generalise these findings to other settings.

While the study period is restricted to 42 months, a declining trend in annual mortality is observed and some of this effect may be due to a number of changes to cART policy in South Africa in recent years, with patients initiated onto cART at higher CD4 counts [36]. It is important that this trend continues to be measured and future studies should evaluate this over a longer duration.

More extensive HIV testing is required in the community so that HIV-positive individuals are identified early and placed on cART prior to developing TB. While mortality in the younger population is much lower than the older population, this study supports the implementation of HIV prevention interventions. Death among younger TB patients could be prevented as the majority of younger TB patients would not have died without HIV. Furthermore, the increased risk of death associated with HIV in patients with TB could be mitigated by knowledge of HIV status and early ART especially for those with low CD4 counts. A recent study demonstrated significant advantages associated with widespread implementation of WHO standards with improved outcomes when HIV-positive patients were started on ART early [37]. Finally, ongoing improvements in data recording and reporting will allow us to continue to assess the epidemic as it evolves.

## Abbrevaitions

cART: Combination antiretroviral therapy
CI: Confidence intervals
HIV: Human immunodeficiency virus
PHC: Primary health care
RR: Relative risk
TB: Tuberculosis
WHO: World Health Organization
